# An Automated CAD System for Accurate Grading of Uveitis Using Optical Coherence Tomography Images

**DOI:** 10.3390/s21165457

**Published:** 2021-08-13

**Authors:** Sayed Haggag, Fahmi Khalifa, Hisham Abdeltawab, Ahmed Elnakib, Mohammed Ghazal, Mohamed A. Mohamed, Harpal Singh Sandhu, Norah Saleh Alghamdi, Ayman El-Baz

**Affiliations:** 1 Electronics and Communications Engineering Department, Mansoura University, Mansoura 35516, Egypt; sshaggag@gmail.com (S.H.); mazim12@mans.edu.eg (M.A.M.); 2 Bioengineering Department, University of Louisville, Louisville, KY 40292, USA; fakhal01@louisville.edu (F.K.); hisham.abdeltawab@louisville.edu (H.A.); aaelna02@louisville.edu (A.E.); harpal.sandhu@gmail.com (H.S.S.); 3 Electrical and Computer Engineering Department, Abu Dhabi University, Abu Dhabi 59911, United Arab Emirates; mohammed.ghazal@adu.ac.ae; 4 College of Computer and Information Science, Princess Nourah Bint Abdulrahman University, Riyadh 11564, Saudi Arabia; nosalghamdi@pnu.edu.sa

**Keywords:** U-NET, deep learning, uveitis grading, OCT segmentation

## Abstract

Uveitis is one of the leading causes of severe vision loss that can lead to blindness worldwide. Clinical records show that early and accurate detection of vitreous inflammation can potentially reduce the blindness rate. In this paper, a novel framework is proposed for automatic quantification of the vitreous on optical coherence tomography (OCT) with particular application for use in the grading of vitreous inflammation. The proposed pipeline consists of two stages, vitreous region segmentation followed by a neural network classifier. In the first stage, the vitreous region is automatically segmented using a U-net convolutional neural network (U-CNN). For the input of U-CNN, we utilized three novel image descriptors to account for the visual appearance similarity of the vitreous region and other tissues. Namely, we developed an adaptive appearance-based approach that utilizes a prior shape information, which consisted of a labeled dataset of the manually segmented images. This image descriptor is adaptively updated during segmentation and is integrated with the original greyscale image and a distance map image descriptor to construct an input fused image for the U-net segmentation stage. In the second stage, a fully connected neural network (FCNN) is proposed as a classifier to assess the vitreous inflammation severity. To achieve this task, a novel discriminatory feature of the segmented vitreous region is extracted. Namely, the signal intensities of the vitreous are represented by a cumulative distribution function (CDF). The constructed CDFs are then used to train and test the FCNN classifier for grading (grade from 0 to 3). The performance of the proposed pipeline is evaluated on a dataset of 200 OCT images. Our segmentation approach documented a higher performance than related methods, as evidenced by the Dice coefficient of 0.988 ± 0.01 and Hausdorff distance of 0.0003 mm ± 0.001 mm. On the other hand, the FCNN classification is evidenced by its average accuracy of 86%, which supports the benefits of the proposed pipeline as an aid for early and objective diagnosis of uvea inflammation.

## 1. Introduction

In recent years, retinal imaging techniques have been greatly exploited by researchers to detect diseases that may cause vision loss. Particularly, optical coherence tomography (OCT) is a popular noninvasive technique that used for diagnosis and assessment of several retinal and corneal diseases [1,2]. Here, we are interested in vitreous inflammation diagnostic and grading [3]. Developing an accurate grading system for vitreous inflammation severity is clinically essential since the vitreous inflammation is considered an important medical diagnostic sign of uveitis. This paper proposes a fully automated computer aided diagnostic (CAD) system for grading of vitreous inflammation, based on extracting discriminatory features from the segmented vitreous regions of OCT images.

Uveitis [4,5,6] is generally a group of intraocular inflammatory diseases that may affect the uvea or destroy the eye tissues. It may affect all ages especially 20 to 60 years and it may last for short time (acute) or long time (chronic). It may be caused by diseases occurring in the eye or it can be part of an inflammatory diseases affecting other parts of the body. It may be infectious or autoimmune in origin. Uveitis may be classified more specifically according to the eye region that is affected by the inflammation into four types: (1) anterior uveitis, which refers to the inflammation affecting the anterior chamber of the eye; (2) intermediate uveitis, if the vitreous is affected; (3) posterior uveitis affecting the back of the eye, retina, and choroid; and (4) panuveitis when all eye major parts are affected: The vitreous inflammation grading is an important and critical target since its almost entirely subjective. Vitreal inflammation presents on examination as a haziness of the vitreous because protein and inflammatory cells leak into the vitreous. There are generally 6 grades of inflammation (0, 0.5, 1, 2, 3, 4) (but grade 4 cannot be assessed because it is not possible to get any clear OCT image from it).

In the literature, using deep learning in integration with neural networks can optimize solutions to several complex problems of classification [7]. machine and deep learning techniques show a potential to perform efficient segmentation of medical structures from OCT images and/or the classification and grading of OCT images [8,9,10,11,12,13,14]. For example, Pelosini et al. [8] developed a segmentation technique that based on a linear regression model, which has lower performance in pathological scans as compared to normal scans [15]. The computer aided diagnostic system of Eltanboly et al. [9] was designed to identify early signs of diabetic retinopathy; however, preprocessing and complicated computations are required for the segmentation of retina layers. Rossant et al. [13] succeeded to to segment the eight retina layers using a hybrid approach incorporating clustering, filtering, and both random field and active contour models. However, their proposed pipeline failed in segmentation of blurred images. Yazdanpanah et al. [14] used active contour energy minimization along with shape priors to segment retina layers. Of note, this was a very early study in the field of spectral-domain optical coherence tomography (SD-OCT), and worked on scans of rat retina obtained using custom hardware. Also, manual segmentation had to be initialized by the user. Haggag et al. [16] developed an automatic U-net convolutional neural network (U-CNN) for segmentation of the vitreous from OCT scans, where the input OCT images were directly applied to train the U-CNN. Their results showed the potential of using U-CNN to solve the problem of vitreous segmentation, but this technique has failed to segment most of severe inflammation images [16]. However, there are still many challenges to get perfect segmentation or classification in some cases of hardly separable images [8,9,15,16].

In trying to develop an automatic system for quantitative assessment of vitreous inflammation, Invernizzi et al. [17] described the inflammatory cells that appear in OCT scans as a hyper reflective dots. Vitreous haze, which may be indicative of inflammation, is also detectable by observing the variations in brightness of vitreous. Pearse et al. [18] developed an automatic technique to quantify the vitreous signal intensity from OCT scans. However, it has a significant limitation as an automatic system since it depends and needs manual segmentation. Schlegl et al. [19] utilized the U-CNN to develop a full automated pipeline to identify and then quantify the intra-retinal cystoid fluid and subretinal fluids.

Many studies used CNN and U-Net to improve OCT segmentation. Cecilia et al. [20] used a U-Net architecture for delineation of macular edema. This technique has achieved an acceptable accuracy which is evaluated by Dice metric of 0.91. He et al. [21] examined the performance of the U-Net architecture relative to a Random Forest-based approach. Finally, Leyuan et al. [22] identified the OCT layer boundaries by mixing the CNN with a graph based method.

**Figure 1:**
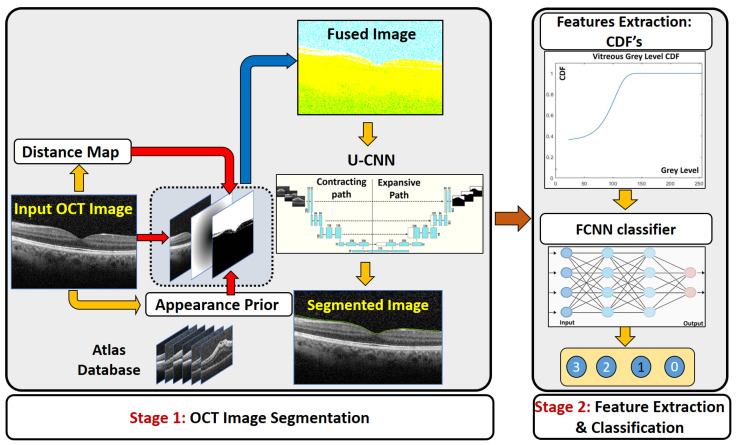
Illustration of the proposed CAD. The first stage is the segmentation stage depending on fused image and U-CNN. The second stage is the classifier to predict the grade of vitreous inflammation severity.

To avoid the aforementioned limitations, the proposed CAD system is divided into two main stages (see Figure 1). The first stage segments the vitreous region using a U-net convolutional neural network aided with using the fused images as a training and testing dataset rather than using the original grayscale images directly in training and testing. The fused images are used as an auxiliary, pre-processing technique. This is followed by a grading stage that is conducted using a machine learning-based classifier into one of five grades (0, 0.5, 1, 2 and 3), where 0 refers to normal vitreous and 3 refers to the worst case of vitreous inflammation. The main contributions of this work are as follows:

- In contrast to [16], where the OCT images were directly applied to train the U-CNN, the first stage of the proposed CAD system trains the U-CNN model using a fused image (FI) dataset, which integrates the information of the original image with a proposed distance map, and a proposed adaptive appearance map (AAP), instead of the direct original images.
- Compared to previous work, the first stage of the proposed CAD system shows superior performance in vitreous segmentation from the OCT images in spite of the great similarity between the vitreous and the background.
- The second stage of the proposed CAD system shows great performance in classification accuracy in spite of the great overlap among the extracted features from the OCT vitreous images.

The rest of the paper is organized as follows. Section 2 details the methods used for the framework itself and for evaluating its accuracy in segmentation as well as classification. Section 3 discusses the experimental results and its details. Experimental results will show the potential of the U-CNN training using the proposed FI dataset to significantly improve the segmentation performance, evidenced by the obtained higher Dice similarity coefficient (DC) metric and the lower Hausdorff distance (HD). The results of vitreous inflammation grading using FCNN is also reported. Finally, Section 4 concludes the paper.

## 2. Materials and Methods

We developed a CAD system for accurate grading of vitreous inflammation from OCT images. The proposed pipeline is composed of two main stages. The first stage is to segment the vitreous region to simplify the processing in the next stage, i.e., grading of the inflammation severity. In our analysis, the total number of used images is 200 OCT of five different grades of severity (grades “0” through “3”). The details of each stage are described in the following subsections.

### 2.1. Segmentation Stage

The first stage of the CAD system is to extract the vitreous region from the images to be ready for accurate grading in the next stage. The segmentation stage is composed of two processes: the construction of a fused image and the application of the U-net CNN. The details of each are described as follows.

#### 2.1.1. Construction of the Fused Image

Due to the similar visual appearance of the vitreous region and other tissues in the background, our pipeline extract different image descriptors from the OCT image to guide the U-CNN segmentation. The extracted image features are integrated with the original OCT intensity image to construct a three-layer image (called the fused image as shown in Figure 1) that is then used for CNN training and testing. The first layer of the fused image consists of the original grayscale OCT image. Since the vitreous region is typically located in the upper part of a given OCT image, we added in the second layer a distance-based image descriptor. Namely, the second layer is represented by a distance map for each image pixel with respect to the center of the image. It is measured from the center to encounter the possible rotation of incoming images.

In addition to the grayscale values and the distance-based image descriptor, we also incorporate a learned appearance prior. An adaptive probabilistic map is constructed for each input image to be segmented, using an atlas database. The atlas consists of grayscale OCT data sets (with their respective labels) from different subjects. The labeled data were obtained by manual delineation of the vitreous region by an OCT expert. During the testing phase, the specific appearance prior, $G_{i}$; i=1,2,⋯N, of an input grayscale image is constructed using the visual appearances of both the atlas grayscale images and their labeled images. A sliding window with a variable width (in our experiment below we start from 11 × 11 pixels) is centered at each pixel location in turn within the image to be segmented. The gray level *g* at each pixel location is noted, and an associated probability is computed from the atlas at the corresponding location. To effect this, the system first collects all grayscale values in the interval [g−Δ,g+Δ] within the sliding window across all atlas OCT, along with their corresponding labels. Here Δ is a tunable threshold value that can be varied from 5 to 90. Then the probability assigned to the pixel location is $P=\frac{N_{x}}{N_{t}}$ where $N_{t}$ is the total number of pixels within the given spatial and grayscale bounds, and $N_{x}$ is the number of such pixels that are labeled as vitreous. This process is repeated for all pixel locations in a given image. The whole operation is repeated to compute the probability map for each test and training OCT image.

#### 2.1.2. U-Net Segmentation

**Figure 2:**
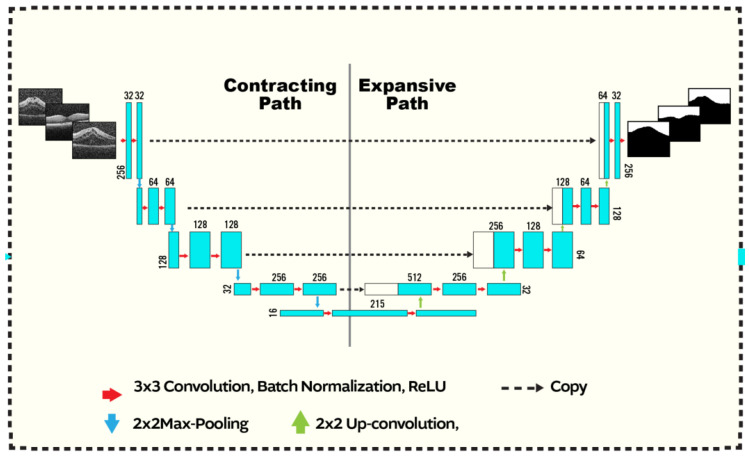
U-CNN structure: The input is a fused image of size 256×256 produced as shown in Figure 1. The segmented output has the same dimensions as the input. Convolution (with a 3×3 kernel), max-pooling, up-convolution operations are respectively indicted using the blue, red, and green arrows. The max-pooling (up-convolution) operation decreases (increases) the spatial dimensions by a factor of 2. The first path starts with 32 kernels and increases up to 512, where it decreases from 512 to 1 in the second. Zero-padding is employed at the boundaries. Copied contextual information afrom the contracting branch are concatenated to the expansive path (dashed arrows).

The second stage of our segmentation pipeline is the U-net CNN, shown in Figure 2. The input to the network is a fused image as constructed above. The U-CNN is composed of two consecutive paths, The first is a contracting path, similar in structure to image classification systems where the fused data are reduced in size and distilled into a set of feature information. The second path is an expansive and up-convolutional network to increase the spatial dimensions and adds context from the second path. Each consecutive block in U-CNN consists of a convolution layer followed by ReLU-activation functions and max pooling process.

The architecture of the contracting or down-sampling section, which increases the number of feature maps, comprises several steps of convolution. Each convolutional block performs two steps of filtering with 3×3 kernels, having unit stride in *x* and *y* directions and ReLU activation functions. Finally, a 2×2 max-pooling is applied at the end of each block. The architecture of the up sampling section is similar to the down sampling section albeit in reversed order as shown.

A sigmoid layer is used at the network output to generate the probabilistic map. Finally, the bipolar cross entropy (BCE) loss function is applied to the network output through training mode, which is computed as

(1) $$L_{BCE}=\sum_{i=1}^{M}-\left(T_{oi}\log\left(P_{oi}\right)+T_{bi}\log\left(P_{bi}\right)\right)$$

where $P_{oi}$, and $P_{bi}$, are the predicted probabilities, computed from the U-net, that a given pixel *i* should be assigned to the vitreous or background segments, respectively. While, $T_{oi}$ ($T_{bi}$) is the ground truth label, i.e., obtained from manual segmentation map, “1” for the object and “0” for the other tissues (vice versa for $T_{bi}$).

### 2.2. Grading Stage

Following the segmentation, the cumulative distribution function (CDF) of grayscale intensities within the segmented region is constructed for each image. These CDF’s are used as the discriminatory features in our machine learning classifier.

For grading, we used a fully connected neural network to classify the vitreous region inflammation into one of five grades (0, 0.5, 1,2 and 3). ’0’ represents the normal eye ’3’ is the is most sever. The FCNN consists of an input layer, 5-nodes output layer, and two hidden layers. The input layer is chosen to be 50 nodes. Each CDF contains 256 points the last 70 points are truncated because all of them is ones and will not discriminate between the different severity degrees. The reminder points are 186 which are used as discriminatory features.

### 2.3. Performance Metrics

Among many different metrics of accuracy, we used the Dice Coefficient of similarity (DC) to measure the accuracy through out the stages of the proposed CAD. Also, we used the Hausdorff Distance (HD) metric to gauge the proximity of the boundary of segmented vitreous to its ground truth counterpart. Let G and S denote the sets of pixels labeled as vitreous in ground truth segmentation and machine segmentation, respectively. The DC metric is defined as follows [23]

(2) $$DC=\frac{2*TP}{2*TP+FN+FP}$$

where TP is the cardinality of the intersection of S and G, FP=|S−G|, and FN=|G−S|. Another performance metric is the Hausdorff distance (HD) that measures the dissimilarity between the boundaries of ground truth and model segmentation. The HD from **G** to **S** is defined as the maximum Euclidean distance d(g,s) between the points *g* from **G** and their closest points *s* in **S**:

(3) $$HD_{G\to S}=\underset{g\epsilon G}{max}\left\{\underset{s\epsilon S}{min}\left\{d\left(g,s\right)\right\}\right\}$$

Note the asymmetry, in that $HD_{G\to S}\neq HD_{S\to G}$ in general. It is easy to define a symmetric version, bidirectional Hausdorff distance $BHD_{G\to S}=max\left\{HD_{G\to S},HD_{S\to G}\right\}$. In order to reduce sensitivity to noise, a further modification is made, replacing the max operation in Equation (3) with taking the 95th percentile.

For the inflammation grading stage evaluation, we computed the average accuracy for all classified grades by the FCNN [24].

(4) $$AverageAccuracy=\sum_{i=1}^{N}\frac{TP_{i}+TN_{i}}{TP_{i}+FN_{i}+FP_{i}}$$

## 3. Experimental Results and Discussions

### 3.1. Data Set

**Figure 3:**
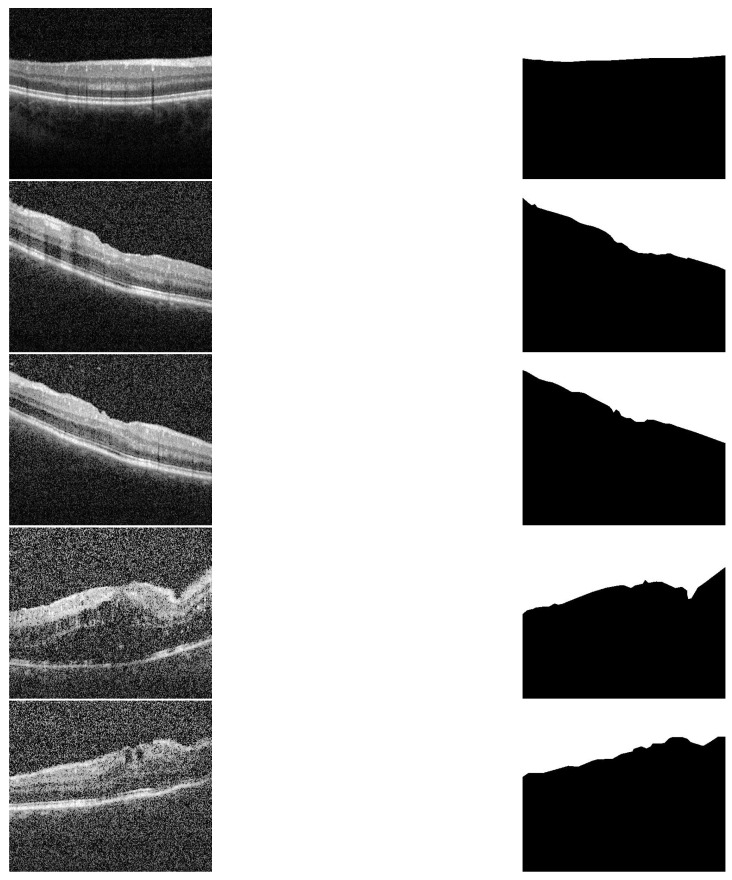
Sample of different grades and their segmentation results. First column (up–to–down) represents the grades 0, 0.5, 1, 2 and 3. The second column represents the corresponding segmentation results, respectively.

The proposed CAD system was applied and tested on 200 OCT images of eyes with different degrees of uveitis severity (0, 0.5, 1, 2, and 3). Sample of all applied inflammation grades and their corresponding segmentation are shown in Figure 3. Imaging was performed with a Spectralis spectral-domain optical coherence tomography (SD-OCT) machine (Heidelberg, Germany) having 4 micron axial resolution and 6 mm × 6 mm in-plane resolution. The rasters comprise thirty horizontal B-scans acquired in order from superior macula to the inferior macula. The entire field of view spans about 20° in both the nasal-temporal and inferior-superior directions, centered on the fovea. Imaging protocol ensured that at least 3 mm of posterior vitreous was visible. The scans that are used in the analysis are selected such that the central horizontal B scan passes through the fovea.

These images were identified using patient database from the uveitis service at the University of Louisville. After the uveitis specialist identified images of patients with uveitis, the images themselves were de-identified for the purpose of analysis. After that, two ophthalmologists graded every image, an attending ophthalmologist with subspecialty expertise and a uveitis fellow (i.e., a fully trained general ophthalmologist who was then undergoing additional subspecialty training in uveitis). They further set together to provide one diagnosis.

The dataset is organized such that 20 images were selected randomly to construct the atlas. The dimensions of the CNN input layer is 256×256, so the images are scaled from its original dimensions of 400×474 to 256×256 to fit the CNN input dimensions. For training and testing, the remainder of the OCT images were partitioned into four groups in order to perform fourfold cross-validation. The accuracy metrics reported are the the average of fourfold results.

### 3.2. Fused Image Construction

Using original gray level images directly in training U-CNN as in [16] results in high accuracy in testing mode but for normal eyes or eyes with low or moderate vitreous haze. But in severe eyes, the vitreous region is very similar to other tissues and hence U-CNN performance is not acceptable. The results of segmentation have many artifacts in either vitreous (false positive) or in other tissues (false negative). To overcome this problem, we propose using the fused images in training and testing rather than the original gray level. The fused image, as explained in Section 2, consists of three layers, original gray level, distance map and appearance prior map (AP). To extract the AP map, an atlas is constructed from 20, randomly selected images that contains all grades of vitreous inflammation. Two parameters control the construction of the atlas which are sliding window width, and Δ. Sliding window is selected on average as 11 × 11 pixels.

**Figure 4:**
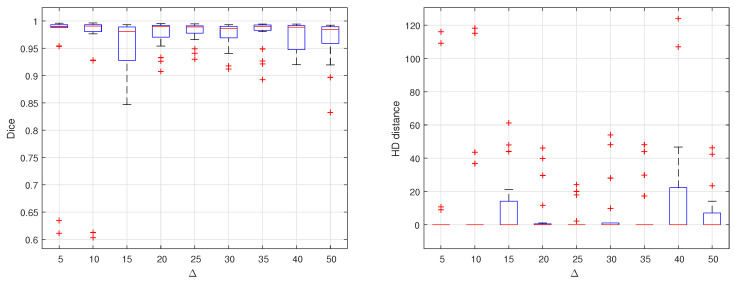
Effect of changing delta on segmentation accuracy evaluated by computing DC and HD distance for different empirically selected values.

The choice of the Δ value has greatly affected the segmentation results. To optimize the value of Δ, many experiments are carried out by different values of Δ. These values range from 5 up to 90. Each time, a complete dataset is produced, trained and tested on the proposed U-Net. Dice similarity and HD are computed for the testing set results. The results are summarized in Figure 4. As shown in the two graphs, it is clear that Δ = 25 results in the optimal performance. Considering the average of DC or HD in each experiment, there is no perceptible difference. But considering all the testing set, the segmentation is performed with almost equal quality at this optimal value of Δ.

### 3.3. Overall Segmentation Evaluation

**Figure 5:**
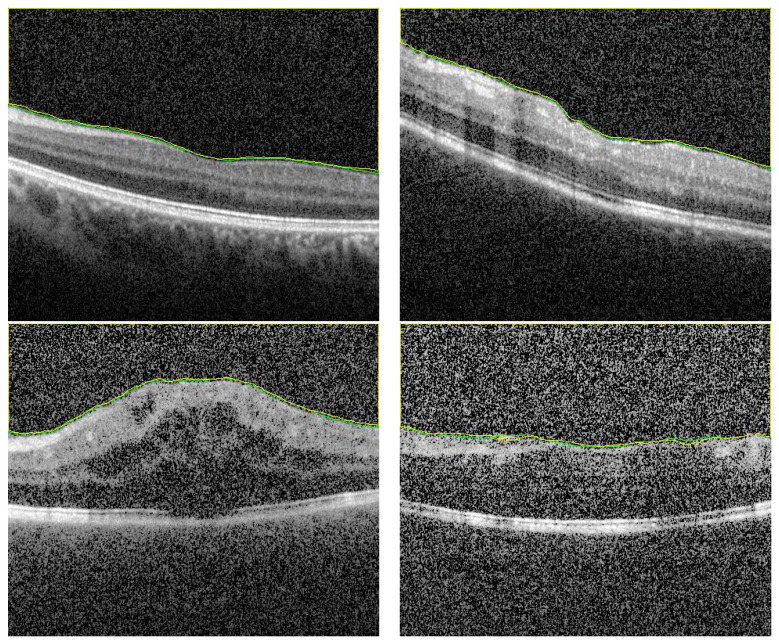
Sample of high accuracy segmentation. The first row represents grayscale images with high contrast, and the second row represents low contrast images with higher degree of vitreous inflammation. Green and yellow colors represents the ground truth and the CNN segmented respectively.

**Figure 6:**
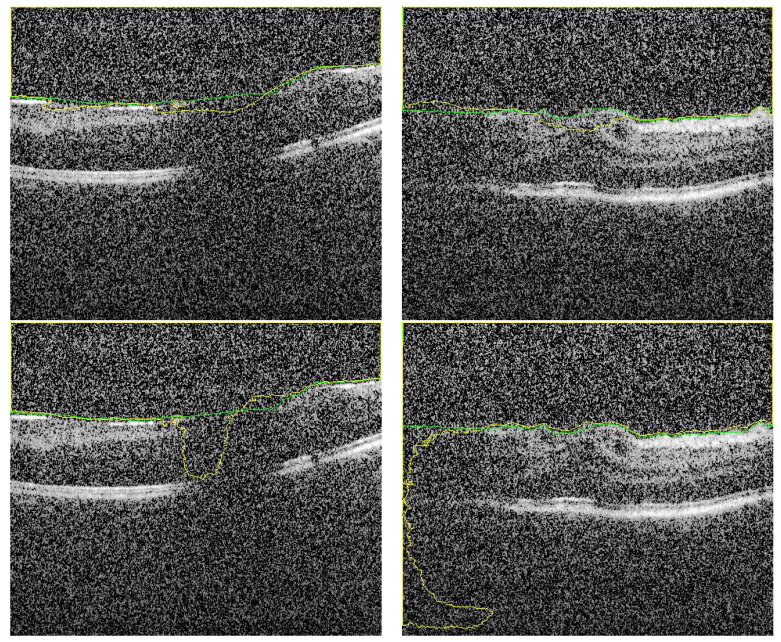
Sample of segmented images of our proposed approach (first row) compared with previous results using only U-Net [16] (second row). The green and yellow colors represents the ground truth and the CNN-segmented respectively.

To demonstrate the accuracy of our approach in the segmentation stage, some representative results are presented in Figure 5 and Figure 6. As demonstrated in from Figure 5, it can be readily seen that the segmentation results have very high accuracy (DC = 0.99) despite the variations in vitreous inflammation degree, which is related to the contrast of the image. In the first row of Figure 5, the image is clear and the contrast between the object and non-object regions is easily spreadable. Although, in the second row, the image has low contrast, the accuracy of the segmentation is nearly the same as that of the first row.

Despite the fact that the selected images in Figure 6 (first row) have low contrast with high similarity between object and non-object regions, our approach has succeeded to segment the vitreous region with high accuracy. The DC for these images ranges from 0.961 to 0.978, which is acceptable but lower accuracy compared to the group of images in Figure 5. However, by visual inspection, the difference between the ground truth (yellow contour) and our system segmentation (green contour) in the first row of Figure 6 is not significant because the region in the middle of the retina is completely unclear. So, the difference between the two contours in the middle region is just a difference between the interpolation capability of two different techniques trying to predict the unclear region. The second row in Figure 6 contains the same images in the first row that with contours resulted from the previous technique. Also, we can confirm that, the high noise in the presented images proves the high efficiency of the proposed technique. Adaptive shape model has highly succeeded to reduce the effect of this noise by selecting proper values of Δ and/or the sliding window size.

To highlight the advantage of the proposed segmentation technique, we compare its performance with segmentation obtained from the previous technique that only utilizes OCT grayscale images [16]. Sample of the compared results are demonstrated in Figure 6 and the summary of the accuracy is given in Table 1. Statistical comparison between the current and previous segmentation methods was carried out using the two-sample Student’s *t*-test. The obtained *p*-values (shown in Table 1) illustrate that there is a statistically significant difference (*p*-value ≤0.05) of the two methods.

### 3.4. Ablation Study

**Figure 7:**
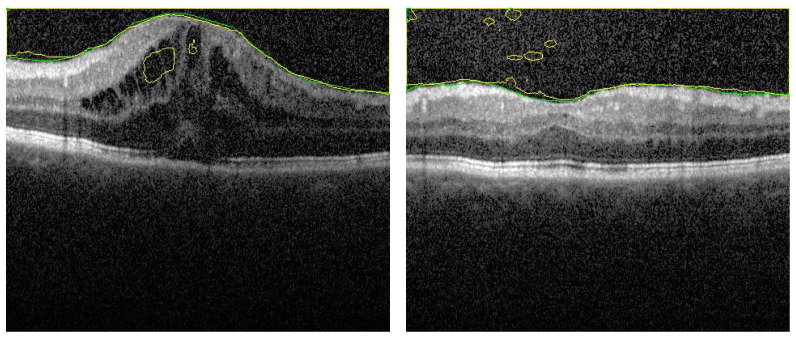
Segmentation sample when AP map is removed from fused images. There are artifacts in both retina layers (**left image**) and vitreous region (**right image**). These artifacts are removed when using the FI as described.

We added here an ablation study for the first stage, segmentation stage, to confirm the validation of our proposal. This study is divided into 2 sections. In the first one, the appearance prior map (AP) is replaced by the gray level in the fused images (FI). That is, the FI contains the distance map and 2 layers of gray level. A fixed number of epochs is maintained in all experiments to justify the results. However about 75% of images is segmented with acceptable accuracy, there are 25% of images still have errors. Sample of images for segmentation is added here to show this effect. In Figure 7 there are some artifacts in the background (FP) due to the similarity of this region in retina with vitreous region (left image). Also, in right image, there are some artifacts in vitreous (FN). These types of errors confirm the importance of the added AP layer to discriminate between the vitreous and the similar tissues in background.

**Figure 8:**
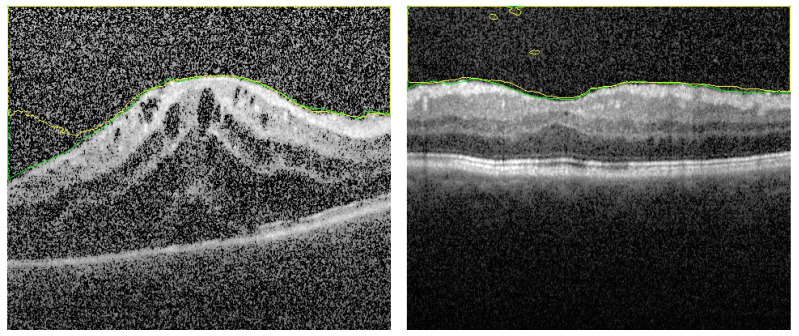
Segmentation sample when distance map is removed from fused images. Number of training epochs is limited. There are some artifacts which are removed when using the FI as described.

**Figure 9:**
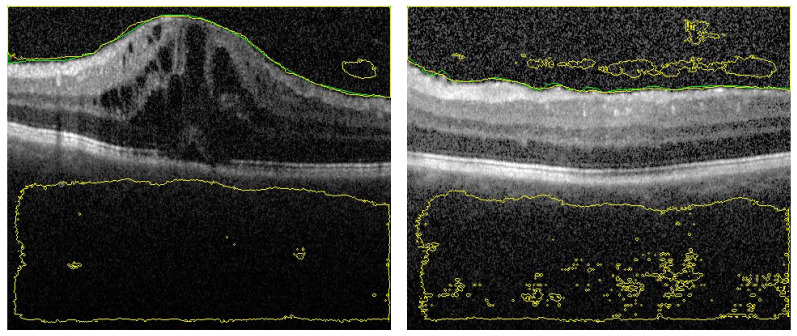
Segmentation sample when distance map is removed from fused images but with greater number of training epochs. There are large segmentation errors.

The second section in the ablation study concerns the effect of removing the distance map from the FI images. The FI images contains the AP map in one layer and 2 layers contain the gray level. In this experiment, about 80% of testing images are segmented with acceptable accuracy. On the other hand, 20% have different types of errors. Sample of results are shown in Figure 8 and in Figure 9 to explain the importance of adding the distance map in our proposal. It is noted that, for limited number of training epochs (above 20 and less than 35) the results of segmentation are acceptable with average DC = 97 ± 2.1%, and the errors of segmentation are limited but still exists. Sample is shown in Figure 8. In trying to improve the accuracy by increasing the number of training epochs, greater errors appear as shown in Figure 9. It is clear that the AP map as well as the distance map increase the stability in training phase, which in turn results in ability to attain high accuracy even in those images of high level of haze.

### 3.5. Grading Stage

Following the segmentation stage, the cumulative distribution function (CDF) of gray scale intensity within the segmented region is constructed for each image of the dataset. The images are categorized into 5 classes according to the vitreous inflammation severity degree as (0, 0.5, 1, 2, 3) grades. Where ‘0’ represents the normal eyes and ‘3’ represents the most severe vitreous inflammation eyes as shown in Figure 3.

In classification process, we carried out many trials with different machine learning techniques to find the most suitable technique for this problem by computing the accuracy in each experiment. The most superior results were from, one hidden layer, fully connected neural networks (FCNN) and from support vector machine (SVM). The highest attained accuracy in SVM trials was 70.1%, and in FCNN trials was 73%. The other techniques results are limited to 53%. The best choice is to improve either SVM or FCNN.

**Figure 10:**
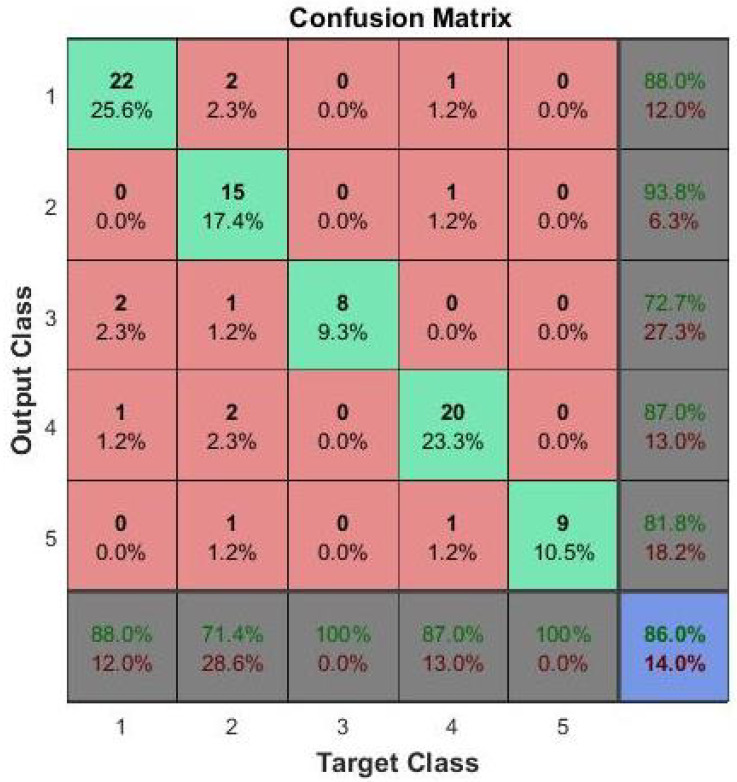
Confusion matrix for the grading details. The classes 1, 2, 3, 4 and 5 correspond to the grades 0, 0.5, 1, 2 and 3 respectively.

The proposed improvement for SVM is to use two level classifier. In the first level, the image is classified as group I or group II: group I has 0 grade, while group II has grades 0.5, 1, 2, and 3. The second stage will discriminate group II into one of the other 4 grades. This technique has greatly improved the accuracy of grading up to 80%. For FCNN, the accuracy is greatly improved by using 2 hidden layers instead of one. Many experiments are carried out with different number of nodes in each layer and the final average accuracy of 86% is obtained.These results the are summarized in Table 2. Depending on the reported results, we selected to use the FCNN as the second stage of the proposed CAD system. A confusion matrix for the testing phase of FCNN results is shown in Figure 10 to clarify the performance of the FCNN in classification of vitreous inflammation grades.

## 4. Conclusions

This paper has introduced a CAD system for vitreous inflammation automatic grading using OCT images. The proposed pipeline is based on a deep learning segmentation approach to extract the vitreous region. Vitreous inflammation severity is assessed by a Fully connected neural network classifier using the CDF of the uveitis intensity which computed from the segmented vitreous. The overall diagnostic accuracy of the proposed pipeline, evaluated using 200 OCT images, supports the benefits of our CAD system as an aid for early and objective diagnosis of uveitis. The proposed technique has proved very high accuracy in the segmentation section depending on the proposed fused images as an input to U-CNN rather than the traditional grey level images. This advantage can be attributed to the integration of appearance prior and distance map with the grey level image. By using this technique, the computational cost is greatly decreased in segmentation process as a result of the great reduction in the number of needed training epochs. One limitation in the grading stage is the very high similarity between the vitreous appearance in different inflammation degrees. This similarity has greatly limited the average accuracy of grading to 86%. In future work, we hope to use more features and to increase the number of images in the data set to improve this value of accuracy.

## Figures and Tables

**Table 1:** Segmentation accuracy comparison of the proposed approach and the previous technique based on U-CNN only [16], using both area and distance-based metrics.

Metrics	This Paper	Haggag et al. [16]	*p*-Value
DC (%)	98.8 ± 1.03	94.0 ± 13.0	≤0.0001
$HD_{95}$ (mm)	0.0003 ± 0.001	0.0360 ± 0.086	≤0.0001

**Table 2:** Grading accuracy comparison of the proposed approach compared with two-level classifier. Here, FCNN and SVM stand for fully connected neural network and support vector machine, respectively.

Metrics	FCNN	Two-Level SVM Classifier
Accuracy (%)	86.0 ± 1.0	80.0 ± 1.0

## Data Availability

Data could be made available after acceptance upon a reasonable request to the corresponding author.
